# Musculoskeletal disorders and discomfort for female surgeons or surgeons with small hand size when using hand-held surgical instruments: a systematic review

**DOI:** 10.1186/s13643-024-02462-y

**Published:** 2024-02-07

**Authors:** Ahmed Basager, Quintin Williams, Rosie Hanneke, Aishwarya Sanaka, Heather M. Weinreich

**Affiliations:** 1 Department of Mechanical and Industrial Engineering, University of Illinois at Chicago, 842 West Taylor Street, Chicago, IL 60607 USA; 2 Department of Industrial Engineering, University of Jeddah, Jeddah, Saudi Arabia; 3 Library of the Health Sciences-Chicago, University of Illinois at Chicago, 1750 W. Polk St, Chicago, IL 60612 USA; 4 Otolaryngology-Head and Neck Surgery, University of Illinois at Chicago, 1009 S. Wood St, Chicago, IL 60612 USA

**Keywords:** Female surgeons, Small-handed surgeons, Musculoskeletal injury, Musculoskeletal disorders, Medical instruments, Glove size, Gender

## Abstract

**Background:**

Work-related musculoskeletal disorders (WMSDs), also referred to as work-related musculoskeletal injuries (MSKIs), cause surgeons pain and discomfort. Implementing ergonomics in the operating room has helped reduce such symptoms. However, there are still many issues that surgeons face when dealing with medical instruments, especially among female surgeons or surgeons with smaller hands.

**Methods:**

The Cochrane methodology for performing a systematic review was utilized to search five databases for pertinent literature based on the study question “Do female surgeons or surgeons with smaller hand size, who use surgical instruments have an increased risk of musculoskeletal disorders and discomfort compared to male or larger handed surgeons?”. The literature search strategy was designed around the three conceptual domains of surgeons/surgery, smaller hand size, and instrumentation. We searched PubMed, Embase.com, CINAHL Plus with Full Text (EBSCOhost), Scopus, and Web of Science Core Collection. This exploration identified 2165 research publications, and after specific inclusion and exclusion criteria, 19 studies were included in the systematic review. Risk of bias analysis was conducted to assess the quality of the included studies. After conducting a heterogeneity test, a meta-analysis was not performed due to high heterogeneity.

**Results:**

Using certain surgical instruments presents challenges in the form of MSKIs for female and smaller-handed surgeons. Studies showed that 77% of females and 73% of surgeons who wear < 6.5 glove size report musculoskeletal issues ranging from difficulty of use to pain. Difficulties using surgical instruments and reported injuries have a greater impact on surgical trainees which might deter interest in surgical fields for future proceduralists. Recommendations for improved ergonomic tool design are suggested by some of the included studies to help tackle the MSKIs that surgeons face when performing operations.

**Conclusions:**

The number of female surgeons has increased substantially in the last decade. Hence, there exists an urgent need to address the major challenges they encounter by focusing on this specific aspect of workplace safety and health to mitigate injury. Doing so will yield a productive environment while simultaneously protecting the health and safety of both surgeons and patients.

**Systematic review registration:**

The study protocol was registered on PROSPERO (ID: CRD42022283378).

**Supplementary Information:**

The online version contains supplementary material available at 10.1186/s13643-024-02462-y.

## Background

Work-related musculoskeletal disorders (WMSD) are injuries or a group of painful disorders of muscles, tendons, skeleton, nerves, and related tissues that occur in work environments that significantly promote the condition or are made worse due to continuous extended activity. Surgery is one of many professions that have this issue. Several studies show that WMSD associated with performing surgery is incredibly common [1, 2]. Gutierrez-Diez et al. [3] showed that 90% of surgeons reported musculoskeletal disorders (MSDs) when performing minimally invasive surgery.

Most common injuries experienced by surgeons occur to the neck (82.9%), lower back (68.1%), shoulder (57.8%), and hands (45%) [4, 5]. To cope with this, surgeons use ergonomic interventions in the operating room, such as adjustable tables and instrument handles near the elbow level, to reduce discomfort and shoulder strain. Other interventions include improved instrument interface, proper monitor placement, adaptable imaging equipment, robotics, camera systems, and integrated hand controls [6–8].

However, one of the main ergonomic obstacles that surgeons face is the hand-held instrument design. Studies show that hand-held surgical instruments may cause musculoskeletal disorders. Specifically, prevalent instrument design issues are associated with laparoscopic surgery [9–11]. Berguer et al. [12] found that in a sample of 149 surgeons, the muscular work of the forearm and thumb muscles is increased when a laparoscopic device is used. In other words, the ergonomic coupling of the surgeon’s hand to the instrument is inadequate due to the handle configuration. Likewise, Trejo et al.’s [13] study revealed that a high proportion of surgeons had concerns such as stiffness, discomfort, and difficulty to perform precise movements when using laparoscopic tools. Twenty-nine percent of surgeons experienced numbness of the fingers or thumb after surgery while 66% faced neck pain when using traditional laparoscopic instruments.

This problem is not limited to laparoscopic or minimally invasive instruments. Surgeons within various surgical specialties (general surgery, plastics, orthopedic, and otolaryngology—head and neck surgery) also report pain and discomfort when using surgical instruments [5, 14–16] and attribute this to the design of the tool. Fram et al. [17] found that 48% of surgeons believe that instruments are not designed for them.

The solution is not simply to build a smaller tool. The majority of surgical instruments are built to perform a specified purpose often with little regard for ergonomics or for the ease of use with which the tools may be handled by the operator, which makes them hard to hold. Rather than the instrument adapting to the operator, the surgeon needs to adapt their operating style, possibly contorting their bodies, to the instrument. Many medical scissors, for example, are constructed with little ergonomic care for comfortable holding; therefore, many surgeons face problems with thumb flexibility and movements when using these types of scissors [5, 14–16].

There are many factors that must be considered when designing a surgical instrument. The design of functional medical devices requires a thorough grasp of human physical capabilities and limitations. A fundamental understanding of the numerous scientific disciplines involved in proper design such as engineering, psychology, anatomy, and physiology is required to build a functional medical device [18].

In this systematic review, the objective was to evaluate the association between sex, hand size, surgical instruments, and MSKIs by answering the following PICO question: “Do female surgeons or surgeons with small hand size, who use surgical hand-held instruments have an increased risk of musculoskeletal disorders and discomfort compared to male or large handed surgeons?”. Specifically, the goal was to evaluate every field of expertise and tool and analyze the instruments that female and small-handed surgeons encounter challenges and musculoskeletal injuries (MSKIs) with.

## Methods

### Search methodology

This study adopts a systematic review methodology to describe and analyze the effects of the use of unfit medical instruments on surgeons. The study protocol was registered on PROSPERO (ID: CRD42022283378) (https://www.crd.york.ac.uk/prospero/). The systematic review was conducted using the Cochrane methodology [19], to ensure a rigorous and comprehensive analysis of the available evidence. Hence, the 2020 Preferred Reporting Items for Systematic Reviews and Meta-Analyses (PRISMA) reporting guideline was followed [20].

### Information sources

Author (R.H.) conducted the database search. The following databases were searched: (1) PubMed, (2) Embase, (3) CINAHL Plus with Full Text (EBSCOhost), (4) Scopus, for an ergonomics and health perspective, and (5) Web of Science Core Collection (including Science Citation Index Expanded, Emerging Sources Citation Index, and Social Sciences Citation Index), for a cross-disciplinary perspective. All databases were searched from inception to 03/17/2023 (Additional file 1).

### Study eligibility criteria, screening, and selection

Inclusion criteria consisted of original, cross-sectional surveys on the ergonomics and human factors of surgical instruments, in relation to hand size, gender, and musculoskeletal symptoms. Publications included from the inception of the selection were required to be written in English and published in scholarly, peer-reviewed journals. Published conference abstracts were excluded, as these abstracts frequently did not get the same level of investigation as original papers. Any articles that had tools that required multiple surgeons to operate were excluded as the focus is on handheld tools by one surgeon. Articles that did not include gender or sex, hand size, or MSKI outcomes were excluded. Finally, articles that had non-handheld tools such as purely robotic instruments were excluded.

One reviewer (A.B.) screened titles and abstracts to identify articles for full-text review. Two reviewers (A.B., H.W.) independently examined the full text of the published journal article. Full-text analysis was carried out to find if the articles pertaining to the information were consistent with the PICO question under consideration. The disputes for article inclusion were settled through consensus-building discussions and, if necessary, a third reviewer (Q.W.) would give the final say.

The following items were abstracted for included articles: title, author names, gender or sex, glove size, medical equipment, medical outcomes, results, population size, medical field, type of article, date of study, age, anthropometric measurements, handiness (use of right or left hand), and time in the operating room.

A test for heterogeneity was conducted and it was found that it had an *I*^2^ of 80% when comparing studies in relation to gender. It also had an *I*^2^ of 83% when comparing results in relation to hand size. Due to the high heterogeneity, we decided that we will not conduct a meta-analysis and that a systemic review would be sufficient.

### Risk of bias

The Checklist for Appraising Surveys tool was used to assess risk of bias in cross-sectional studies [21]. This method included thirteen detailed questions and was introduced in Foster and Jewell [21] *Assembling the pieces of a systematic review: a guide for librarians* book (Additional file 2). This method was used to rate each study as having a low risk of bias, medium risk of bias, or high risk of bias. Two reviewers (A.B., H.W.) did the analysis of the included articles.

## Results

**Fig. 1:**
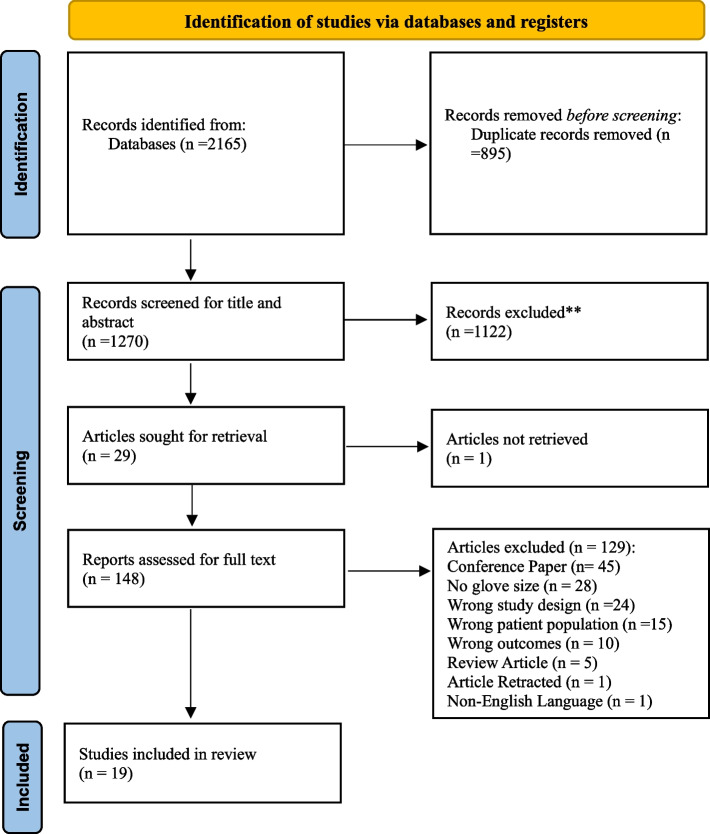
PRISMA flow diagram for systematic review

A total of 2165 articles were identified (PubMed, 361; Embase, 990; CINAHL, 111; Scopus, 442; and Web of Science combined, 261). There were 895 duplicates, and after removal, 1270 scientific papers were included for a title and abstract screening. A total of 1122 articles were excluded, leaving 148 articles for the full-text analysis. An additional 129 articles were excluded that did not meet the criteria, and an additional study was not considered as their full-text paper was unavailable, yielding a total of 19 papers to be included in this review (Fig. 1).

### Risk of bias

The results of the risk of bias are shown in Table 1. Four articles were identified as low risk, 11 articles as medium risk, and 4 articles as high risk.

**Table 1:** Risk of bias results

Article	Count	Risk of bias
Adams et al. (2008) [22]	10.5	Low risk
Berguer and Hreljac (2004) [23]	9	Medium risk
Dabholkar et al. (2017) [24]	6	High risk
Filisetti et al. (2015) [25]	7.5	High risk
Fram et al. (2021) [17]	8.5	Medium risk
Franasiak et al. (2012) [26]	9.5	Medium risk
Gilbert et al. (2013) [27]	10.5	Low risk
Kono et al. (2012) [28]	8.5	Medium risk
Kroon and Fay (2009) [29]	5	High risk
Lucas-Hernandez et al. (2014) [30]	8.5	Medium risk
Morais et al. (2020) [31]	11	Low risk
Park et al. (2010) [32]	9.5	Medium risk
Pawa et al. (2021) [33]	10	Medium risk
Shepherd et al. (2016) [34]	8.5	Medium risk
Sutton et al. (2013) [35]	8	Medium risk
Green et al. (2022) [36]	7.5	High risk
Weinreich et al. (2022) [37]	10.5	Low risk
Yong et al. (2023) [38]	8	Medium risk
Pawa et al. (2022) [39]	9.5	Medium risk

### Study characteristics

**Fig. 2:**
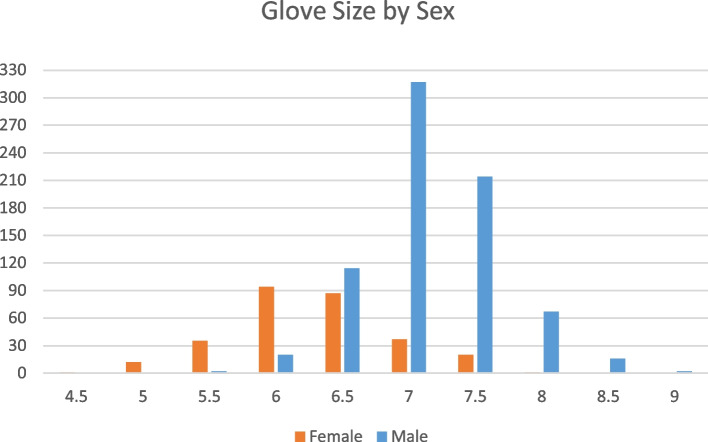
Glove size by sex

The key characteristics of the study are presented in Table 2. All included studies [17, 22–39] were from the past 19 years. The oldest study [23] was from 2004 and the most recent [17] was published in 2023. The sample size for the surgeon population varied across the studies; six studies [22, 24, 29, 34, 36, 38] had a small sample size (*n* ≤ 100), ten studies [17, 25–28, 30–32, 35, 39] had a moderate sample size (between 100 and 400), while three studies [23, 33, 37] had a large sample size (*n* ≥ 400). All studies provided detailed populations based on gender or sex except one [29]. Thirty-one percent of all the surgeons surveyed were female. All studies discuss the glove size of surgeons but only four [17, 23, 25, 30] had detailed descriptions of the glove size for each participant. These detailed glove sizes are shown in Fig. 2.

**Table 2:** Included articles’ key characteristics

First author (year)	Article	Population (gender)	Medical equipment	Measured outcomes
Berguer (2004) [23]	The relationship between hand size and difficulty using surgical instruments: a survey of 726 laparoscopic surgeons	726 (159 female, 567 male)	Laparoscopic instruments (grasper, scissors, dissector, needle, and stapler)	The difficulty of using the instruments. Subjects were grouped as either having MS problems or not having MS problems
Fram (2021) [17]	Female sex is associated with increased reported injury rates and difficulties with use of orthopedic surgical instruments	204 (119 female, 84 male)	Rongeurs, reduction clamps, arthroscope, arthroscopic shaver, mallets, kerrisons, needle drivers, ringed instruments, microsagittal saws, forceps, osteotomes, and burrs	Numbness, stiffness, fatigue, and pain
Filisetti (2015) [25]	Analysis of hand size and ergonomics of instruments in pediatric minimally invasive surgery	138 (33 females, 105 males)	Grasper, scissors, dissector, needle holder, staplers, endobag, clip placement, Ligasure, Ultracision, and endoloops	Musculoskeletal problems (related to arms or back or legs or neck)
Kono (2012) [28]	Rating and issues of mechanical anastomotic staplers in surgical practice: a survey of 241 Japanese gastroenterological surgeons	241 (74 females, 167 males)	Circular and linear staplers	Stress
Lucas-Hernandez (2014) [30]	Ergonomics problems due to the use and design of dissector and needle holder: a survey in minimally invasive surgery	118 (39 females, 79 males)	Laparoscopic instruments (dissector and needle holder)	Fatigue experienced in shoulder-arm, wrist-hand-fingers, neck, back, and elbow-forearm are the most relevant musculoskeletal disorders. Furthermore, paresthesia, pain, and cramps in the wrist-hand fingers
Park (2010) [32]	Patients benefit while surgeons suffer: an impending epidemic	317 (54 females, 261 males)	Standard graspers, needle drivers, energy/coagulation devices, stapler	Physical discomfort or symptoms in the neck, right hand, arm, and lower extremities
Kroon (2009) [29]	Is glove size a predictor for occupational injury in obstetrics and gynecology?	17	Neville-Barnes forceps	Neurapraxia, RMCL thumb, ulna collateral ligaments, extensor compartment, upper arm, shoulder, back and neck, head, ankle
Shepherd (2016) [34]	Ergonomics in laparoscopic surgery—a survey of symptoms and contributing factors	50 (15 females, 35 males)	Johan’s grasper, Maryland’s forceps, scissors, and hook diathermy	Symptoms were reported in at least 1 body region (neck/shoulder, back, hand/wrist, fatigue/irritability)
Adams (2008) [22]	One size does not fit all: current disposable laparoscopic devices do not fit the needs of female laparoscopic surgeons	65 (28 female, 37 males)	Laparoscopic staplers, laparoscopic harmonic scalpel, laparoscopic LigaSure, and laparoscopic retrieval bags	Awkward to use, not easy to use, have to modify hand to use, and use two hands
Dabholkar (2017) [24]	A survey of work-related musculoskeletal disorders among otolaryngologists	73 (27 females, 46 males)	Otoscope, endoscope, drills, suction handles, and microscope	Pain in the upper limbs (elbow, wrist, and hand pain)
Franasiak (2012) [26]	Physical strain and urgent need for ergonomic training among gynecologic oncologists who perform minimally invasive surgery	260 (106 females, 154 males)	Bipolar, monopolar, needle driver, and grasper	Injury, physical strain, and pain
Gilbert (2013) [27]	Ergonomics and bronchoscopy: a survey of the American Association of Bronchology and Interventional Pulmonology	160 (23 females, 137 males)	Endoscopic equipment	Pain location: neck, upper back, lower back, appendicular skeleton, shoulder, arm, wrist, hand, finger
Pawa (2021) [33]	Are all endoscopy-related musculoskeletal injuries created equal? Results of a national gender-based survey	1698 (583 females, 1115 males)	Endoscopes	Injury location: thumb, neck, hand/finger, lower back, shoulder, and wrist
Sutton (2013) [35]	The ergonomics of women in surgery	314 (54 females, 260 males)	Laparoscopic instrument handles	Numbness, stiffness, fatigue, and pain
Morais (2020) [31]	Prevalence, risk factors and global impact of musculoskeletal injuries among endoscopists: a nationwide European study	171 (94 females, 77 males)	Endoscopes	Musculoskeletal injury location: neck pain, thumb pain, wrist pain, and hand numbness
Green (2022) [36]	One size does not fit all: impact of hand size on ease of use of instruments for minimally invasive surgery	58 (17 females, 41 males)	Laparoscopic instruments	Difficulty, fatigue, pressure, loss of strength, and wrist discomfort
Weinreich (2022) [37]	Gender-differences of proceduralists in perception of hand-held surgical instrument fit – a cross-sectional survey	488 (412 females, 76 males)	All types of clamps, Kelly clamp, mosquito, endoscopes, surgical drills, double action scissors, harmonic scalpel, Debakey forceps, laparoscopic, needle drivers, staplers, retractors, LigaSure	Difficult of use and trouble
Yong (2023) [38]	Controller size matters: user proficiency is affected by endoscopic controller size	54 (28 females, 26 males)	Endoscope and bronchoscope	Fatigue
Pawa (2022) [39]	Endoscopy-related injury among gastroenterology trainees	168 (83 female, 85 males)	Endoscope	Thumb pain, hand/finger pain, hand/arm numbness, carpal tunnel syndrome, De Quervain’s tendonitis, wrist pain, elbow pain, shoulder pain, neck pain, upper back pain, lower back pain, hip pain, knee pain, and foot pain

## Discussion

The results from the studies were mostly conclusive. As seen by most authors, medical instrument design is one major reason why female surgeons or surgeons with small hands face musculoskeletal injuries when practicing. The majority of survey surgeon report MSKIs and up to 87% of female surgeons report this has to do with instruments [23]. The authors described musculoskeletal issues that surgeons face differently based on their interests. Most prominently musculoskeletal injuries, pain, and fatigue were addressed the most, followed by the difficulty of use and stress. Medical instruments tested in the studies varied due to different fields of surgery, but mostly laparoscopic and endoscopic instruments had the most focus in the articles.

### Impact of gender or sex

Gender was a big determinant in having musculoskeletal issues. Studies [17, 22, 24, 26–28, 31, 33–36, 38, 39] showed that around 77% of females report musculoskeletal issues ranging from difficulty of use to pain compared to 64% of males. Female surgeons (59 to 100%) compared to male surgeons (13 to 56%) had more problems when using surgical instruments. In all included studies, the average glove size for women (6.0 to 6.5) was significantly smaller than that for males (7.0 to 8.0), which was a big factor in having difficulty in using the instruments, so there is a link between gender and hand size [30]. Women were more likely to describe surgical hand-held instruments as “usually difficult” [22, 36, 37] and requiring the use of two hands [22]. Female surgeons were much more likely to report having negative views regarding orthopedic surgical tools and finding some devices to be challenging or painful to use [17]. Morais, Pawa, and Yong [31, 33, 38] mentioned that women also have a higher risk of MSKI due to differences in hand size and grip power. Endoscopic movements require greater strength and effort on the part of women [33]. This raises the possibility of developing a repetitive strain injury. According to Sutton [35], female surgeons are more likely to treat the hands more than their male counterparts related to doing the same surgical procedures, which include the wrist, thumb, and fingers. Kono et al. [40] dove deeper in another study and stated that since the required operating force exceeds the maximal grip force, it is physically difficult for the majority of Japanese women surgeons to fire the stapler by clutching the proximal side of the lever, which likely explains the tension these women felt and expressed.

### Impact of hand size

Small glove size on its own was also cited as a reason for developing musculoskeletal issues when using surgical hand-held instruments. Studies [22, 23, 28, 31, 34, 37] showed that around 73% of surgeons who wear ≤ 6.5 glove size report musculoskeletal issues ranging from difficulty of use to pain compared to 31% of surgeons who wear a glove size > 6.5. Berguer [23] found that the small glove-size group had a larger percentage of participants who reported difficulty using any laparoscopic device. Shepard [34] also indicated that people with gloves smaller than 6.5 were more likely to report moderate to severe symptoms connected to handle dimensions and more likely to have worse symptoms connected to laparoscopic surgery activities.

### Prevalence and type of instruments not fitting

Twelve of the articles included detailed reported problems when using surgical hand-held instruments. For example, Kono [28], Berguer [23], Weinreich [37], and Adams [22] all mentioned that the current design of the stapler causes pain and discomfort for surgeons especially female and small glove-handed surgeons. As a result, it was found that instrument use was a problem for females more than for males. These three studies [22, 23, 28] suggest that between 78 and 92% of females deem the stapler as hard to use compared to 41–56% of males. Filisetti [25] showed that the needle holder had a greater difficulty score in all hand-size groups. Morais [31], Pawa [33], and Yong [38] reported that around 72.5% of endoscopists faced at least one musculoskeletal injury due to the use of endoscopes. Moreover, Shepherd [34], Sutton [35], and Green [36] discussed the unfit use of some of the laparoscopic instruments that caused moderate to serious symptoms ranging from discomfort to back and neck problems.

However, in three of the included studies, there seems to be a high level of satisfaction with the fit of some of the surgical instruments that have been studied. Franasiak [26] reported instrument fit as “just right” for bipolar devices, graspers, and monopolar devices. Park [32] also mentions high percentage levels of instrument handle size being adequate for graspers, laparoscopic needle drivers, and staplers. Lucas-Hernandez [30] had results showing mixed reviews of the laparoscopic dissector but a high level of satisfaction for the laparoscopic needle holder. To clarify, these results are specific only to the fit and handle size of some of the mentioned instruments. Strength nor power or posture needed to use was discussed. Franasiak [26] reported a very high 88.1% of strain among the participating surgeons of the frequently used laparoscopic devices. Lucas-Hernandez [30] reported that 68% of surgeons take uncomfortable or forced postures when using the laparoscopic dissector and 61% reported they take an uncomfortable or forced posture when using the laparoscopic needle holder.

Nevertheless, there is minimal discussion of the biological factors between men and women with the same hand size in the included studies. Only Sutton [35] discussed the differences between both sexes with the same hand size; the author explained that women with big or small hand size reported more problems than men with the same glove size. This may be due to different factors but it certainly has to be investigated more. It is an area of study that is slightly neglected but exposed that the problems that face women when using medical instruments are more than just a difference in size problems.

### Effects on training

Trainees also reported having problems when using surgical hand-held instruments. Kroon [29] stated that female trainees reported injuries to their dominant hand. Morais [31] also mentioned that 78.2% of surgeons believed that hand size affected endoscopy learning. Moreover, Pawa [39] found that half of the participants in a national survey of gastroenterology trainees reported having at least one endoscopic-related injury (ERI) and findings imply that certain ERI vulnerabilities manifest during training and should be checked. Improving these medical tools to accommodate more female surgeons and those with small hands will help in attracting more of these trainees into surgical professions as some might have decided against certain medical specialties due to the discomfort they had when practicing with these types of equipment.

### Ergonomic suggestions

Three of the studies included in this systemic review suggest that improvements should be made to surgical hand-held instruments to help in lowering musculoskeletal issues and some propose ways on how to ergonomically improve some of the instruments. For instance, practitioners in the Shepherd [34] study suggested that rotating mechanisms, smaller handle dimensions, and softer handles as improvements to laparoscopic instruments. Lucas-Hernandez proposed enhancing the laparoscopic dissector and needle holder’s design mechanism to make it easier to use by making it lighter while keeping or boosting the sensitivity of the distal surgical instrument. Furthermore, 79% of those polled for Park’s [32] article claimed that they would use different-length instruments if they were available.

### Strengths and limitations

This systematic review has several strengths. First, after a lengthy literature search, it is determined that this is the only review that has been done that tackles specifically the issue of difficulty of hand-held surgical instruments in relation to the combination of gender, sex, and hand size. Second, this study’s methodology was thorough. Moreover, the inclusion of detailed glove size analysis and instrument analysis in this study, along with a critical appraisal of the existing literature, helps to highlight the unique contributions of this study compared to previous systematic reviews [41, 42]. By carefully examining and evaluating the limitations or gaps in the existing literature, we were able to identify areas where this study can make a significant contribution to the field.

On the other side, there are certain limitations to this systematic review. There was significant heterogeneity among the studies in terms of definitions of MSKIs and thus precluded a meta-analysis. Additionally, terms used such as discomfort, pain, and difficulty of use are subjective. Furthermore, there is significant bias in how populations were selected for surveys and many of the studies did not publish methodology that was clear enough for reproducibility. Further research examining the impact of MSKI needs to move beyond subjective assessment and provide objective measurements that can then be compared across studies. Furthermore, it must be mentioned that 18 of the included studies that discussed hand-size dealt with glove size and not anthropometric hand measurement sizes. These glove sizes are determined by surgeons themselves, which may lead to some surgeons wearing bigger or smaller glove sizes.

While recognizing the limitations of this systematic review, one can observe that the papers included in this review span a period of 19 years. It is important to understand that, despite the advancements in technology and medicine over this period, many of the medical instruments included are still the ones that are used today. Even though some of the included studies were carried out on older dates, they can nevertheless provide insightful information. Additionally, the fact that fairly recent publications still report problems using the same tools as old published journal articles suggests a persistent issue that has not dissipated over the 19-year span. Notably, comparing the oldest paper [23] in the review with one of the newest [36] reveals a noteworthy observation: both studies highlight issues with the same laparoscopic tools, indicating that despite potential advancements, certain challenges persist in the use of these instruments.

## Conclusion

As this systematic review points out, many female and small hand surgeons are having major issues when dealing with medical instruments. Thus, there is a need to develop new ergonomic designs for some of the surgical hand-held instruments that surgeons use most often. The number of female surgeons has risen substantially in the last decade, so there is an imperative need to address the major challenges that they face when operating. Having a productive environment through a complete health and safety evaluation of the design and potential redesign of medical instruments will not only reflect positively on the surgeon’s health and ability but also could create scenarios for better patient health outcomes as well.

### Supplementary Information

**Additional file 1.** Search strategy.

**Additional file 2.** The Checklist for Appraising Surveys tool was used to assess risk of bias in cross-sectional studies  [21].

## Data Availability

Database search method will be provided in an additional file.

## Abbreviation

PICO: Problem/Population, Intervention, Comparison, Outcome
PRISMA: Preferred Reporting Items for Systematic Reviews and Meta-Analyses
PROSPERO: International Prospective Register of Systematic Reviews
PubMed: Public/Publisher MEDLINE
CINAHL: Cumulative Index to Nursing and Allied Health Literature
Embase: Excerpta Medica Database
Scopus: Elsevier’s abstract and citation database

## References

[1] Voss RK, Chiang Y, Cromwell KD, Urbauer DL, Lee JE, Cormier JN, Stucky CH (2017). Do no harm, except to ourselves? A survey of symptoms and injuries in oncologic surgeons and pilot study of an intraoperative ergonomic intervention. J Am Coll Surg.

[2] Aaron KA, Vaughan J, Gupta R, Ali N, Beth AH, Moore JM, Ma Y, Ahmad I, Jackler RK, Vaisbuch Y (2021). The risk of ergonomic injury across surgical specialties. PLoS One.

[3] Gutierrez-Diez MC, Benito-Gonzalez MA, Sancibrian R, Gandarillas-Gonzalez MA, Redondo-Figuero C, Manuel-Palazuelos JC (2018). A study of the prevalence of musculoskeletal disorders in surgeons performing minimally invasive surgery. Int J Occup Saf Ergonomics.

[4] Szeto GP, Ho P, Ting AC, Poon JT, Cheng SW, Tsang RC (2009). Work-related musculoskeletal symptoms in surgeons. J Occup Rehabil.

[5] Soueid A, Oudit D, Thiagarajah S, Laitung G (2010). The pain of surgery: pain experienced by surgeons while operating. Int J Surg.

[6] Berquer R, Smith WD, Davis S (2002). An ergonomic study of the optimum operating table height for laparoscopic surgery. Surg Endosc Other Interv Tech.

[7] Vereczkel A, Bubb H, Feussner H (2003). Laparoscopic surgery and ergonomics: it’s time to think of ourselves as well. Surg Endosc Other Interv Tech.

[8] Miller K, Benden M, Pickens A, Shipp E, Zheng Q (2012). Ergonomics principles associated with laparoscopic surgeon injury/illness. Hum Factors.

[9] Nguyen NT, Ho HS, Smith WD, Philipps C, Lewis C, De Vera RM, Berguer R (2001). An ergonomic evaluation of surgeons’ axial skeletal and upper extremity movements during laparoscopic and open surgery. Am J Surg.

[10] Sari V, Nieboer TE, Vierhout ME, Stegeman DF, Kluivers KB (2010). The operation room as a hostile environment for surgeons: physical complaints during and after laparoscopy. Minim Invasive Ther Allied Technol.

[11] Tung KD, Shorti RM, Downey EC, Bloswick DS, Merryweather AS (2015). The effect of ergonomic laparoscopic tool handle design on performance and efficiency. Surg Endosc.

[12] Berguer R, Forkey DL, Smith WD (1999). Ergonomic problems associated with laparoscopic surgery. Surg Endosc.

[13] Trejo AE, Doné KN, DiMartino AA, Oleynikov D, Hallbeck MS (2006). Articulating vs. conventional laparoscopic grasping tools—surgeons’ opinions. Int J Ind Ergonomics..

[14] Catanzarite T, Tan-Kim J, Whitcomb EL, Menefee S (2018). Ergonomics in surgery: a review. Female Pelvic Med Reconstr Surg.

[15] Matern U, Koneczny S (2007). Safety, hazards and ergonomics in the operating room. Surg Endosc.

[16] Berguer R (1999). Surgery and ergonomics. Arch Surg.

[17] Fram B, Bishop ME, Beredjiklian P, Seigerman D (2021). Female sex is associated with increased reported injury rates and difficulties with use of orthopedic surgical instruments. Cureus.

[18] Weinger MB, Wiklund ME, Gardner-Bonneau DJ. Handbook of human factors in medical device design. Boca Raton: CRC Press; 2010.

[19] Higgins JP, Thomas J, Chandler J, Cumpston M, Li T, Page MJ, Welch VA. Cochrane handbook for systematic reviews of interventions. Hoboken: Wiley; 2019. 10.1002/14651858.ED000142 PMC10284251 31643080

[20] Page MJ, McKenzie JE, Bossuyt PM, Boutron I, Hoffmann TC, Mulrow CD, Shamseer L, Tetzlaff JM, Akl EA, Brennan SE (2021). The PRISMA 2020 statement: an updated guideline for reporting systematic reviews. Syst Rev.

[21] Foster MJ, Jewell ST (2017). Assembling the pieces of a systematic review: a guide for librarians.

[22] Adams DM, Fenton SJ, Schirmer BD, Mahvi DM, Horvath K, Nichol P (2008). One size does not fit all: current disposable laparoscopic devices do not fit the needs of female laparoscopic surgeons. Surg Endosc.

[23] Berguer R, Hreljac A (2004). The relationship between hand size and difficulty using surgical instruments: a survey of 726 laparoscopic surgeons. Surg Endosc Other Interv Tech.

[24] Dabholkar T, Yardi S, Dabholkar YG, Velankar HK, Ghuge G (2017). A survey of work-related musculoskeletal disorders among otolaryngologists. Indian J Otolaryngol Head Neck Surg.

[25] Filisetti C, Cho A, Riccipetitoni G, Saxena AK (2015). Analysis of hand size and ergonomics of instruments in pediatric minimally invasive surgery. Surg Laparosc Endosc Percutan Tech.

[26] Franasiak J, Ko EM, Kidd J, Secord AA, Bell M, Boggess JF, Gehrig PA (2012). Physical strain and urgent need for ergonomic training among gynecologic oncologists who perform minimally invasive surgery. Gynecol Oncol.

[27] Gilbert CR, Akulian JA, Feller-Kopman D, Yarmus L (2013). Ergonomics and bronchoscopy: a survey of the American Association of Bronchology and Interventional Pulmonology. J Bronchol Interv Pulmonol.

[28] Kono E, Tomizawa Y, Matsuo T, Nomura S (2012). Rating and issues of mechanical anastomotic staplers in surgical practice: a survey of 241 Japanese gastroenterological surgeons. Surg Today.

[29] Kroon ND, Fay HR (2008). Is glove size a predictor for occupational injury in obstetrics and gynaecology?. J Obstet Gynaecol.

[30] Lucas-Hernández M, Pagador JB, Pérez-Duarte FJ, Castelló P, Sánchez-Margallo FM (2014). Ergonomics problems due to the use and design of dissector and needle holder: a survey in minimally invasive surgery. Surg Laparosc Endosc Percutan Tech.

[31] Morais R, Vilas-Boas F, Pereira P, Lopes P, Simões C, Dantas E, Cunha I, Roseira J, Cortez-Pinto J, Silva J (2020). Prevalence, risk factors and global impact of musculoskeletal injuries among endoscopists: a nationwide European study. Endosc Int Open.

[32] Park A, Lee G, Seagull FJ, Meenaghan N, Dexter D (2010). Patients benefit while surgeons suffer: an impending epidemic. J Am Coll Surg.

[33] Pawa S, Banerjee P, Kothari S, D’Souza SL, Martindale SL, Gaidos JK, Oxentenko AS, Burke CA (2021). Are all endoscopy-related musculoskeletal injuries created equal? Results of a national gender-based survey. J Am Coll Gastroenterol..

[34] Shepherd JM, Harilingam MR, Hamade A (2016). Ergonomics in laparoscopic surgery—a survey of symptoms and contributing factors. Surg Laparosc Endosc Percutan Tech.

[35] Sutton E, Irvin M, Zeigler C, Lee G, Park A (2014). The ergonomics of women in surgery. Surg Endosc.

[36] Green SV, Morris DE, Naumann DN, Rhodes HL, Burns JK, Roberts R, Lang AR, Morris L. One size does not fit all: impact of hand size on ease of use of instruments for minimally invasive surgery. Surgeon. 2022;21(5):267–72. 10.1016/j.surge.2022.11.001 36513570

[37] Weinreich HM, Babu M, Kamil R, Williams Q, Buhimschi IA (2022). Gender-differences of proceduralists in perception of hand-held surgical instrument fit–a cross-sectional survey. Am J Surg.

[38] Yong V, Kahler D, Schlossberg A, Gilmore K, Zhao H, Philp MM (2023). Controller size matters: user proficiency is affected by endoscopic controller size. Am J Surg.

[39] Pawa S, Martindale SL, Gaidos JK, Banerjee P, Kothari S, D’Souza SL, Oxentenko AS, Burke CA (2022). Endoscopy-related injury among gastroenterology trainees. Endosc Int Open.

[40] Kono E, Tada M, Kouchi M, Endo Y, Tomizawa Y, Matsuo T, Nomura S (2014). Ergonomic evaluation of a mechanical anastomotic stapler used by Japanese surgeons. Surg Today.

[41] Barnard E, Sheaffer K, Hampton S, Measel ML, Farag A, Shaw C (2021). Ergonomics and work-related musculoskeletal disorders: characteristics among female interventionists. Cureus.

[42] Bellini MI, Amabile MI, Saullo P, Zorzetti N, Testini M, Caronna R, D’Andrea V (2022). A woman’s place is in theatre, but are theatres designed with women in mind? A systematic review of ergonomics for women in surgery. J Clin Med.

