# Supplementary material for: Musculoskeletal disorders and discomfort for female surgeons or surgeons with small hand size when using hand-held surgical instruments: a systematic review
Source: Syst Rev. 2024 Feb 7;13:57. doi: 10.1186/s13643-024-02462-y (PMC10848514; doi:10.1186/s13643-024-02462-y)
Supplement: Supplementary file 1 — Additional file 1. Search strategy. [file 13643_2024_2462_MOESM1_ESM.docx]

Additional file .1 Search strategy:

| **Database** | **Search string** |
| --- | --- |
| PubMed | (("glove size*"[tiab] OR "hand size*"[tiab] OR "small hand"[tiab] OR "smaller hand"[tiab] OR "small hands"[tiab] OR "smaller hands"[tiab] OR "large hand"[tiab] OR "larger hand"[tiab] OR "large hands"[tiab] OR "larger hands"[tiab]) OR (("Ergonomics"[mh:noexp] OR "Hand"[mesh] OR "Hand Strength"[mesh] OR ergonomic*[tiab] OR grip*[tiab] OR pinch*[tiab] OR grasp*[tiab]) AND ("Physicians, Women"[mesh] OR "Sex Factors"[mesh] OR "Sex Characteristics"[mesh] OR "sex differences"[tiab] OR "gender differences"[tiab] OR "sex factors"[tiab] OR "sex characteristics"[tiab] OR "women surgeon*"[tiab] OR "woman surgeon*"[tiab] OR "female surgeon*"[tiab] OR "female operator*"[tiab] OR "female physician*"[tiab] OR "woman physician*"[tiab] OR "women physician*"[tiab] OR gender[tiab] OR sex[tiab] OR demographic*[tiab]))) AND ("Equipment Design"[mesh] OR "Laparoscopes"[mesh] OR "Surgical Instruments"[mesh] OR "Disposable Equipment"[mesh] OR "instrumentation"[subheading] OR "endoscopes"[mesh] OR equipment[tiab] OR device*[tiab] OR drill*[tiab] OR tool*[tiab] OR instrument*[tiab] OR stapler*[tiab] OR scissors[tiab] OR laparoscope*[tiab] OR needle*[tiab] OR clamp*[tiab] OR scalpel*[tiab] OR "forceps"[tiab] OR hemostat*[tiab] OR clip*[tiab] OR "ligasure"[tiab] OR "ultracision"[tiab] OR clip*[tiab] OR endobag*[tiab] OR endoscope*[tiab] OR "trocar"[tiab] OR "trocars"[tiab] OR "suture"[tiab] OR "sutures"[tiab]) AND ("Laparoscopy"[mesh] OR "Endoscopy"[mesh] OR "Specialties, Surgical"[mesh] OR surgery[tiab] OR surgeries[tiab] OR surgeon*[tiab] OR surgical[tiab] OR laparoscop*[tiab] OR endoscop*[tiab]) |
| Embase.com | (('glove size*':ti,ab OR 'hand size*':ti,ab OR 'small hand':ti,ab OR 'smaller hand':ti,ab OR 'small hands':ti,ab OR 'smaller hands':ti,ab OR 'large hand':ti,ab OR 'larger hand':ti,ab OR 'large hands':ti,ab OR 'larger hands':ti,ab) OR (('ergonomics'/exp OR 'hand'/exp OR 'hand strength'/exp OR ergonomic*:ti,ab OR grip*:ti,ab OR pinch*:ti,ab OR grasp*:ti,ab) AND ('female physician'/exp OR 'sex factor'/exp OR 'sexual characteristics'/exp OR 'sex differences':ti,ab OR 'gender differences':ti,ab OR 'sex factors':ti,ab OR 'sex characteristics':ti,ab OR 'women surgeon*':ti,ab OR 'woman surgeon*':ti,ab OR 'female surgeon*':ti,ab OR 'female operator*':ti,ab OR 'female physician*':ti,ab OR 'woman physician*':ti,ab OR 'women physician*':ti,ab OR gender:ti,ab OR sex:ti,ab OR demographic*:ti,ab))) AND ('equipment design'/exp OR 'surgical equipment'/exp OR 'laparoscope'/exp OR 'disposable equipment'/exp OR 'device comparison'/lnk OR 'endoscope'/exp OR equipment:ti,ab OR device*:ti,ab OR drill*:ti,ab OR tool*:ti,ab OR instrument*:ti,ab OR stapler*:ti,ab OR scissors:ti,ab OR laparoscope*:ti,ab OR needle*:ti,ab OR clamp*:ti,ab OR scalpel*:ti,ab OR forceps:ti,ab OR hemostat*:ti,ab OR clip*:ti,ab OR ligasure:ti,ab OR ultracision:ti,ab OR endobag*:ti,ab OR endoscope*:ti,ab OR trocar:ti,ab OR trocars:ti,ab OR suture:ti,ab OR sutures:ti,ab) AND ('laparoscopy'/exp OR 'endoscopy'/exp OR 'surgery'/exp OR surgery:ti,ab OR surgeries:ti,ab OR surgeon*:ti,ab OR surgical:ti,ab OR laparoscop*:ti,ab OR endoscop*:ti,ab) |
| CINAHL Plus with Full Text | ((TI ( "glove size*" OR "hand size*" OR "small hand" OR "smaller hand" OR "small hands" OR "smaller hands" OR "large hand" OR "larger hand" OR "large hands" OR "larger hands" ) OR AB ( "glove size*" OR "hand size*" OR "small hand" OR "smaller hand" OR "small hands" OR "smaller hands" OR "large hand" OR "larger hand" OR "large hands" OR "larger hands" ) ) OR (((MH "Ergonomics") OR (MH "Hand+") OR (MH "Grip Strength") OR TI ( ergonomic* OR grip* OR pinch* OR grasp* ) OR AB ( ergonomic* OR grip* OR pinch* OR grasp* ) ) AND ((MH "Physicians, Women") OR (MH "Sex Factors") OR TI ( "sex differences" OR "gender differences" OR "sex factors" OR "sex characteristics" OR "women surgeon*" OR "woman surgeon*" OR "female surgeon*" OR "female operator*" OR "female physician*" OR "woman physician*" OR "women physician*" OR gender OR sex OR demographic* ) OR AB ( "sex differences" OR "gender differences" OR "sex factors" OR "sex characteristics" OR "women surgeon*" OR "woman surgeon*" OR "female surgeon*" OR "female operator*" OR "female physician*" OR "woman physician*" OR "women physician*" OR gender OR sex OR demographic* )))) AND ((MH "Equipment Design+") OR (MH "Surgical Instruments") OR (MH "Disposable Equipment") OR (MH "Endoscopes+") OR (TI (equipment OR device* OR drill* OR tool* OR instrument* OR stapler* OR scissors OR laparoscope* OR needle* OR clamp* OR scalpel* OR "forceps" OR hemostat* OR clip* OR "ligasure" OR "ultracision" OR clip* OR endobag* OR endoscope* OR "trocar" OR "trocars" OR "suture" OR "sutures")) OR (AB (equipment OR device* OR drill* OR tool* OR instrument* OR stapler* OR scissors OR laparoscope* OR needle* OR clamp* OR scalpel* OR "forceps" OR hemostat* OR clip* OR "ligasure" OR "ultracision" OR clip* OR endobag* OR endoscope* OR "trocar" OR "trocars" OR "suture" OR "sutures"))) AND ((MH "Laparoscopy") OR (MH "Endoscopy+") OR (MH "Surgery, Operative+") OR (MH "Specialties, Surgical+") OR ( TI (surgery OR surgeries OR surgeon* OR surgical OR laparoscop* OR endoscop*)) OR (AB (surgery OR surgeries OR surgeon* OR surgical OR laparoscop* OR endoscop*))) |
| Web of Science, includes: Science Citation Index Expanded, Emerging Sources Citation Index, Social Sciences Citation Index, and Conference Proceedings Citation Index | ((TS=((("glove size*" OR "hand size*" OR "small hand" OR "smaller hand" OR "small hands" OR "smaller hands" OR "large hand" OR "larger hand" OR "large hands" OR "larger hands") OR (("Hand Strength" OR ergonomic* OR grip* OR pinch* OR grasp*) AND ("Sex Factors" OR "Sex Characteristics" OR "sex differences" OR "gender differences" OR "women surgeon*" OR "woman surgeon*" OR "female surgeon*" OR "female operator*" OR "female physician*" OR "woman physician*" OR "women physician*" OR gender OR sex OR demographic*))) )) AND TS=(("Surgical Instruments" OR "instrumentation" OR equipment OR device* OR drill* OR tool* OR instrument* OR stapler* OR scissors OR laparoscope* OR needle* OR clamp* OR scalpel* OR "forceps" OR hemostat* OR clip* OR "ligasure" OR "ultracision" OR clip* OR endobag* OR endoscope* OR "trocar" OR "trocars" OR "suture" OR "sutures") )) AND TS=((surgery OR surgeries OR surgeon* OR surgical OR laparoscop* OR endoscop*)) |
| Scopus | ( ( TITLE-ABS-KEY ( "glove size*" OR "hand size*" OR "small hand" OR "smaller hand" OR "small hands" OR "smaller hands" OR "large hand" OR "larger hand" OR "large hands" OR "larger hands" ) OR TITLE-ABS-KEY ( ( "Hand Strength" OR ergonomic* OR grip* OR pinch* OR grasp* ) AND ( "Sex Factors" OR "Sex Characteristics" OR "sex differences" OR "gender differences" OR "women surgeon*" OR "woman surgeon*" OR "female surgeon*" OR "female operator*" OR "female physician*" OR "woman physician*" OR "women physician*" OR gender OR sex OR demographic* ) ) ) ) AND ( TITLE-ABS-KEY ( "Surgical Instruments" OR "instrumentation" OR equipment OR device* OR drill* OR tool* OR instrument* OR stapler* OR scissors OR laparoscope* OR needle* OR clamp* OR scalpel* OR "forceps" OR hemostat* OR clip* OR "ligasure" OR "ultracision" OR clip* OR endobag* OR endoscope* OR "trocar" OR "trocars" OR "suture" OR "sutures" ) ) AND ( TITLE-ABS-KEY ( surgery OR surgeries OR surgeon* OR surgical OR laparoscop* OR endoscop* ) ) |
