# Supplementary material for: Musculoskeletal disorders and discomfort for female surgeons or surgeons with small hand size when using hand-held surgical instruments: a systematic review
Source: Syst Rev. 2024 Feb 7;13:57. doi: 10.1186/s13643-024-02462-y (PMC10848514; doi:10.1186/s13643-024-02462-y)
Supplement: Supplementary file 2 — Additional file 2. The Checklist for Appraising Surveys tool was used to assess risk of bias in cross-sectional studies [21]. [file 13643_2024_2462_MOESM2_ESM.docx]

Additional File .2 The Checklist for Appraising Surveys tool was used to assess risk of bias in cross-sectional studies^22^:

| ***Quality Indicators*** | Yes | No | Unclear | N/A |
| --- | --- | --- | --- | --- |
| 1. Did the study address a clearly focused research question? |  |  |  |  |
| 2. Is the research method (study design) appropriate for answering the research question? |  |  |  |  |
| 3. Is the method of selection of participants (patients, students, faculty, customers) clearly described? |  |  |  |  |
| 4. Is the sample of participants representative with regard to the population to which the findings will be generalized? |  |  |  |  |
| 5. Could the way the sample was obtained introduce potential (selection) bias? |  |  |  |  |
| 6. Was a pilot version of a survey administered to participants representative of those in the sampling frame, and the instrument modified accordingly? |  |  |  |  |
| 7. Was the survey used in the study likely to be valid and reliable? |  |  |  |  |
| 8. If the study compares different subgroups from the survey, were the data obtained using the same methods from these different groups? |  |  |  |  |
| 9. Was the response rate adequate? |  |  |  |  |
| 10. Have non-responders been accounted for? |  |  |  |  |
| 11. Was the statistical analysis appropriate (e.g., statistical analysis for quantitative answers, and qualitative analysis for open-ended questions)? |  |  |  |  |
| 12. Is there an adequate description of the data? |  |  |  |  |
| 13. Have all relevant results (“significant” and “nonsignificant”) been reported ? |  |  |  |  |
